# Long term trends of breast cancer incidence according to proliferation status

**DOI:** 10.1186/s12885-022-10438-1

**Published:** 2022-12-21

**Authors:** Elise Klæstad, Signe Opdahl, Sunil Xavier Raj, Anna Mary Bofin, Marit Valla

**Affiliations:** 1 Department of Clinical and Molecular Medicine, Faculty of Medicine and Health Sciences, Norwegian University of Science and Technology, Trondheim, Norway; 2 Department of Public Health and Nursing, Faculty of Medicine and Health Sciences, Norwegian University of Science and Technology, Trondheim, Norway; 3 Cancer Clinic, St. Olav’s Hospital, Trondheim University Hospital, 7006 Trondheim, Norway; 4 Department of Pathology, St. Olav’s Hospital, Trondheim University Hospital, 7006 Trondheim, Norway; 5 Clinic of Laboratory Medicine, St. Olav’s Hospital, Trondheim University Hospital, 7006 Trondheim, Norway

**Keywords:** Breast cancer, Incidence trends, Proliferation, Ki-67, Mitotic count

## Abstract

**Background:**

Long-term breast cancer incidence trends according to proliferation status are poorly described. We studied time-trends in breast cancer incidence, using mitotic count and Ki-67 as markers of proliferation.

**Methods:**

Among 83,298 Norwegian women followed for breast cancer occurrence 1961–2012, 2995 incident breast cancers were diagnosed. Ki-67 was assessed using immunohistochemistry on tissue microarrays and mitoses were counted on whole sections. We compared incidence rates according to proliferation status among women born 1886–1928 and 1929–1977, estimating age-specific incidence rate ratios. We performed multiple imputations to account for unknown proliferation status. Mean values of Ki-67 and mitotic counts were calculated, according to age and birth year. We performed separate incidence analyses for HER2^+^ and triple negative breast cancers.

**Results:**

Among women aged 40–69 years, incidence rates of tumours with low-proliferative activity were higher among those born in 1929 or later, compared to before 1929, according to Ki-67 and mitotic count. Incidence rates of tumours with high-proliferative activity were also higher in women born in 1929 or later compared to before 1929 according to Ki-67, but not according to mitotic count. Mean values of Ki-67 and mitotic count varied according to age and birth year. In subtype-specific analyses we found an increase of high-proliferative HER2^+^ tumours according to Ki-67 in women born in 1929 or later, compared to before 1929.

**Conclusions:**

There has been a temporal increase in both low- and high-proliferative breast cancers.

**Supplementary Information:**

The online version contains supplementary material available at 10.1186/s12885-022-10438-1.

## Introduction

In Norway, breast cancer incidence rates have doubled since the establishment of the Cancer Registry of Norway 60 years ago [1]. Breast cancer mortality rates remained stable from 1965 until 1995 but have shown a steady decline from 1995 until today. Nevertheless, breast cancer is the second most common cause of cancer related deaths among Norwegian women [1]. Worldwide, breast cancer is the most common cancer and the leading cause of cancer related death among women [2]. The discovery of the molecular subtypes in the 2000s [3] gave new insight into the heterogeneity of breast cancer. These subtypes are associated with different risk factors [4–6], incidence trends [7], prognosis [7, 8] and treatment response [9]. The molecular subtypes of breast cancer are defined by gene expression patterns [3, 10]. Molecular subtyping can also be done using immunohistochemistry (IHC) and in situ hybridization (ISH) as surrogates for gene expression analyses [11–13]. Studies of incidence trends have demonstrated an increase in oestrogen receptor (ER)^+^ breast cancers [7, 14–18], and a decrease in ER^−^ tumours [15–18]. The increase in ER^+^ tumours, and the decrease in ER^−^ tumours has been described for all ages [16–18]. There has also been an increase in luminal A [7, 15, 19] and luminal B (Human epidermal growth factor receptor 2 (HER2)^−^) breast cancers [7]. The increase in ER^+^ tumours has been attributed in part to the use of menopausal hormone therapy (MHT) and the introduction of mammography screening programs [20–22].

Proliferation is one of the hallmarks of cancer [23–25]. High proliferation is associated with poor prognosis in breast cancer [26–28], and can be measured using mitotic count, Ki-67 protein expression and gene expression assays [11, 29–32]. High mitotic counts and Ki-67 levels are associated with reduced overall survival and disease free survival [33]. Ki-67 can be used to select early-stage breast cancer patients for chemotherapy and to monitor treatment response [34, 35]. Recently, adjuvant therapy with abemaciclib in combination with endocrine therapy was approved by the Food and Drugs Administration (FDA) in the USA as a treatment option in high-risk, hormone receptor positive, HER2 negative, node-positive, early breast cancers with Ki-67 ≥ 20% [36]. In addition, Ki-67 can be used to separate ER^+^/HER2^−^ tumours into luminal A and luminal B (HER2^−^) [12, 35]. Due to interobserver and interlaboratory variations and lack of consensus on cut-off levels, the clinical use of Ki-67 has been debated [32, 34, 35]. According to Norwegian guidelines, mitotic count is also routinely reported as a marker of tumour cell proliferation [32].

Several studies have described time trends in incidence of breast cancer overall and according to hormone receptor status and molecular subtype, but time trends according to proliferation status remain largely unknown. Such knowledge may improve our understanding of the natural course of breast cancer and stimulate hypotheses on aetiology and prevention, in particular when combined with knowledge on other societal changes. Our aim was to study long-term trends in incidence of high- and low-proliferative breast cancers in a population of Norwegian women born between 1886 and 1977, using mitotic counts and Ki-67 as markers of proliferation.

## Materials and methods

This follow-up study comprises three large cohorts of Norwegian women who were followed for breast cancer occurence. Information regarding incident breast cancers was obtained from the Cancer Registry of Norway using national identity numbers to link person data. The breast cancers that occurred among these women have previously been characterized by our group, and they were reclassified into histological grade and molecular subtypes [7, 8, 37]. As part of histological grading [38, 39], mitotic counts were registered for all available tumours, and as part of molecular subtyping, Ki-67 positive cells were counted in 500 tumour cells. Mitotic counts and Ki-67 levels were also available for some of the breast cancers that were not successfully reclassified into molecular subtypes. More details are provided below.

### Cohort 1

Between 1956 and 1959 all women (*n* = 25,727) in the northern part of Trøndelag County, Norway, born between 1886 and 1928 were invited to attend a clinical screening for early detection of breast cancer [40]. These women were followed for breast cancer occurrence from January 1st, 1961, until the date of breast cancer diagnosis, death from other causes, emigration, or December 31st, 2008 [8]. Among these women, 1379 incident breast cancer cases were registered. After diagnosis, patients were followed until death from breast cancer or from other causes, or until December 31st, 2010. Of the 1379 incident breast cancers, 909 were previously reclassified into molecular subtypes [8]. Mitotic counts were missing for 466 of the 1379 cases, and Ki-67 status was missing for 496 cases.

### Cohort 2

Between 1995 and 1997, all women in the northern part of Trøndelag County, Norway, born between 1897 and 1977 were invited to participate in the HUNT2 study [41]. A total of 34,221 women were followed for breast cancer occurrence from attendance until the date of breast cancer diagnosis, death from other causes, emigration, or December 31st, 2009 [7]. Among these, 731 incident breast cancer cases were registered. After diagnosis, patients were followed until death from breast cancer or other causes, or until December 31st, 2015. Of the 731 cases, 653 have previously been reclassified into molecular subtypes [7, 8]. Mitotic counts were missing for 77 of the 731 cases, and Ki-67 levels were missing for 84 cases.

### Cohort 3

All women born at E. C. Dahl’s foundation in Trondheim (in the southern part of Trøndelag) between 1920 and 1966 were followed for breast cancer occurrence from January 1st, 1961, until the date of breast cancer diagnosis, death from other causes, emigration, or December 31st, 2012 [42]. Of the 23,350 women included, 885 incident breast cancer cases were registered [37]. Participants were followed until death from breast cancer or any other causes, or until December 31st, 2015. Of the 885 cases, 545 have previously been reclassified into molecular subtypes [37]. Mitotic counts were missing for 343 of the 885 cases, and Ki-67 levels were missing for 340 cases.

### Specimen characteristics

In previous work, chromogenic in situ hybridization (CISH), fluorescence in situ hybridization (FISH) and IHC on tissue micro arrays (TMAs) were used to classify tumours into molecular subtypes according to the algorithm presented in Table 1 [7, 8, 37] . For TMA construction, three 1-mm-in-diameter tissue cores were taken from the tumour periphery. According to current guidelines, Ki-67 was counted in 500 epithelial tumour cells in hot spot areas and reported as the proportion of nuclei with positive IHC staining [32]. Scoring and reporting of the other molecular markers used for molecular subtyping have previously been described in detail [7, 8, 37]. All IHC markers were assessed by two independent observers.

**Table 1:** Reclassification of breast cancers into molecular subtype

Molecular subtype	Molecular marker
Luminal A	ER^+^ and/or PR^+^, HER2^−^, Ki-67 < 15%
Luminal B (HER2^−^)	ER^+^ and/or PR^+^, HER2^−^, Ki-67 ≥ 15%
Luminal B (HER2^+^)	ER^+^ and/or PR^+^, HER2^+^
HER2 type	ER^−^, PR^−^, HER2^+^
Basal phenotype	ER^−^, PR^−^, HER2^−^, CK5^+^ and/or EGFR^+^
5 negative phenotype	ER^−^, PR^−^, HER2^−^, CK5^−^, EGFR^−^

Abbreviations: *ER* oestrogen receptor, *PR* progesterone receptor, *HER2* human epidermal growth factor receptor 2, *CK5* cytokeratin 5, *EGFR* epidermal growth factor receptor

In the present study we pooled data from the three cohorts. In the analysis of incidence rates, we used data from a total of 2995 breast cancers that occurred among 83,298 women. Participants in the three cohorts were included from parts of the county of Trøndelag and across an overlapping range of birthyear (Supplementary Fig. 1). Thus, some women were included in more than one cohort. By using a case specific identity number, 171 incident breast cancer cases overlapping between two or all three cohorts were identified (5.7%). Due to anonymization of study participants, identification of overlap in the healthy background population was not possible. Therefore, to avoid underestimation of incidence rates we did not exclude duplicate incident cancers in the analyses.

Mean values of Ki-67 was estimated based on the results from two independent observers. In cases with only one Ki-67 assessment, this was used. Tumours were then subdivided into categories according to Ki-67 status. We used two different cut-off levels for Ki-67: a) </≥15%, and b) </≥30% positive cells.

Mitoses were previously counted manually by two independent pathologists in ten high power fields in whole sections of breast cancer [7, 8, 37]. In the present study, mitotic counts from the two observers were recalculated to number of mitoses/mm^2^ [32, 39] and mean values were calculated. In cases with only one observation, this was used. According to WHO guidelines for histological grading mitotic counts are assigned a score from 1 to 3 based on thresholds for mitoses/mm^2^ [32, 39]. Based on these thresholds, we used two different cut-off levels for mitotic count: a) ≤/> 3.6 mitoses/mm^2^ (mitotic score 1 versus mitotic score 2 and 3), and b) </≥7.7 mitoses/mm^2^ (mitotic score 1 and 2 versus mitotic score 3).

### Statistical analyses

In the incidence analyses, we used the same cut-off for birth cohort as in a previous study by our group [7], and separated the study population into two groups: women born before 1929 and women born in 1929 or later. Incidence rates for all cancers combined and for each category of Ki-67 and mitotic count were calculated and plotted according to birth year and age at diagnosis. Poisson regression was used to compare incidence rates between women born before 1929 and women born in 1929 or later.

To examine Ki-67 and mitotic count as continuous variables, mean values with standard deviation (SD) were calculated according to age groups and birth year.

To examine whether a change in proliferation status over time occurred within specific molecular subtypes, we also analysed incidence rates according to birth cohort, Ki-67 status, and mitotic counts in HER2^+^ and Triple negative (TN) tumours. Due to limited statistical power, these analyses were restricted to women aged 50 to 80 years. The TN tumours comprised all basal phenotype (BP) and 5-negative phenotype (5NP) tumours, and the HER2^+^ tumours comprised the HER2 type and luminal B (HER2^+^) subtypes. Separate analyses were not performed for luminal A and luminal B (HER2^−^) tumours as they were already defined by Ki-67 status.

For some breast cancer cases, Ki-67 and/or mitotic count was unavailable. Ki-67 status was missing in 920 (31%) of the tumours, and mitotic count was missing in 886 (30%) of the tumours. Supplementary Table 1 gives an overview of tumour characteristics of cases with and without missing Ki-67 values. To compensate for missing values, multiple imputations [43, 44] were used to predict mitotic count and Ki-67 status for these cases. The imputation model included all information available: age (5-year categories) and calendar year at diagnosis (continuous), stage (I-IV, unknown), extent of disease (localized to the breast, local invasion, regional lymph nodes, distant lymph nodes or organ metastases, metastases detected, unknown) as reported by the Cancer Registry of Norway, year of birth (5-year categories), follow-up time after diagnosis (log transformed, continuous) and survival status (alive, death from breast cancer, death from other causes), with the assumption that data were missing at random [44]. Incidence rates and incidence rate ratios (IRR) with 95% confidence intervals (CI) according to birth year and age were calculated based on 50 imputed datasets. STATA version 17 (STATA Corp.) was used for statistical analyses.

## Results

### Characteristics of study population

Mean age at baseline was 51.0 for women in cohort 1, 50.1 years for women in cohort 2 and 21.2 for women in cohort 3 (Table 2). Mean follow-up for breast cancer occurrence in the three cohorts was 29.7, 12.5 and 37.4 years, respectively. Mean age at diagnosis was 70.7 in cohort 1, 63.2 in cohort 2 and 54 years in cohort 3. Of the cases with known Ki-67 status, 538 (60%), 391 (60%) and 268 (50%) cases had Ki-67 < 15%, in cohorts 1 to 3, respectively. Furthermore, 120 (14%), 108 (17%) and 123 (23%) cases had Ki-67 ≥ 30% in the three cohorts, respectively. Of the cases with known mitotic count, 539 (61%) in cohort 1, 428 (65%) in cohort 2 and 334 (61%) in cohort 3 had mitotic counts ≤3.6. Furthermore, 147 (16%), 97 (15%) and 92 (17%) cases had mitotic counts ≥7.7, respectively.

**Table 2:** Characteristics of the study populations used in estimation of breast cancer incidence

Women followed for breast cancer occurrence	Cohort 1	Cohort 2	Cohort 3
	Women born 1886–1928	Women born 1897–1977	Women born 1920–1966
Number of women	25,727	34,221	23,350
Mean age at baseline ^a^ (SD)	51.0 (11.6)	50.1 (17.5)	21.2 (3.7)
Mean follow up for BC occurrence ^b^ (SD)	29.7 (13.9)	12.5 (2.7)	37.4 (9.1)
Women with incident breast cancer			
Number of cases	1379	731	885
Mean age at diagnosis (SD)	70.7 (11.8)	63.2 (13.7)	54 (10.3)
Mean follow up after diagnosis (SD)	9.1 (8.9)	9.5 (4.8)	10.8 (7.5)
Death from breast cancer (%)	612 (44)	123 (17)	173 (20)
Death from other causes (%)	688 (50)	152 (21)	81 (9)
Ki-67/500 tumour cells (%)			
Cases with missing status	496 (36)	84 (11)	340 (38)
< 15	538 (39)	391 (53)	268 (30)
≥ 15, < 30	225 (16)	148 (20)	154 (17)
≥ 30	120 (9)	108 (15)	123 (14)
Mitotic count (mitoses/mm^2^) (%) ^c^			
Cases with missing status	466 (34)	77 (11)	343 (39)
≤ 3.6	539 (39)	428 (58)	334 (38)
> 3.6, < 7.7	227 (16)	129 (18)	116 (13)
≥ 7.7	147 (11)	97 (13)	92 (10)
Molecular subtypes (%)			
Luminal A	433 (31)	354 (48)	236 (27)
Luminal B (HER2-)	248 (18)	157 (21)	178 (20)
Luminal B (HER2+)	71 (5)	48 (7)	65 (7)
HER2 type	62 (4)	33 (5)	24 (3)
5NP	33 (2)	19 (3)	6 (1)
BP	62 (4)	42 (6)	36 (4)
Unknown	470 (34)	78 (11)	340 (38)
Stage (%) ^c^			
I	671 (49)	388 (53)	278 (31)
II	483 (35)	288 (39)	220 (25)
III	93 (7)	30 (4)	26 (3)
IV	116 (8)	25 (3)	31 (4)
Unknown	16 (1)	0	330 (37)
Extent of disease (%) ^c^			
Disease localized to the breast	501 (36)	372 (51)	422 (48)
Local invasion	42 (3)	12 (1)	3 (0)
Regional lymph nodes	363 (27)	219 (30)	303 (34)
Distant lymph node or organ metastases	99 (7)	23 (3)	31 (4)
Metastases detected, unknown location	2 (0)	0	0
Unknown	372 (27)	105 (14)	126 (14)

^a)^ At time of entry

^b^ For women who were included prior to 20 years of age, follow up for breast cancer diagnosis started at their 20th birthday

^c^ As recorded by the Cancer registry of Norway. Information is based on histopathological and/or clinical examination

Abbreviations: *SD* standard deviation; *BC* breast cancer; *5NP* 5 negative phenotype; *BP* basal phenotype

### Age-specific incidence rates according to year of birth

For women aged 40 to 69 years, age-specific breast cancer incidence rates were higher in women born in 1929 or later compared to those born before 1929 (Table 3).

**Table 3:** Incidence rates, incidence rate differences (IRD) and incidence rate ratios (IRR) of proliferation markers Ki-67 and mitotic count according to age at diagnosis and year of birth

		Observed					Imputed^a^				
		Incidence rate (cases/100000 person-years)					Incidence rate (cases/100000 person-years)				
	Age	Women born before 1929	Women born in 1929 or later	IRD	IRR	(95% CI)	Women born before 1929	Women born in 1929 or later	IRD	IRR	(95% CI)
Total^b^	40–49	78.3	127.4	49.1	1.6	(1.3–2.1)					
	50–59	110.0	214.4	104.4	1.9	(1.6–2.3)					
	60–69	170.6	298.5	127.9	1.7	(1.5–2.0)					
	70–79	246.5	240.6	−5.9	1.0	(0.8–1.2)					
Ki-67/500 tumour cells (%)											
< 15	40–49	16.9	34.6	17.7	2.1	(1.2–3.4)	31.9	50.2	18.3	1.6	(1.0–2.5)
	50–59	21.8	90.1	68.3	4.1	(2.9–5.9)	49.8	124.2	74.4	2.5	(1.9–3.3)
	60–69	58.9	146.3	87.4	2.5	(2.0–3.1)	92.3	186.7	94.4	2.0	(1.6–2.5)
	70–79	112.5	121.7	9.2	1.1	(0.8–1.5)	151.2	147.2	−4	1.0	(0.7–1.3)
≥15	40–49	15.9	51.5	35.6	3.2	(1.9–5.4)	46.1	77.1	31.0	1.7	(1.2–2.4)
	50–59	31.0	62.3	31.3	2.0	(1.5–2.8)	60.1	90.2	30.1	1.5	(1.1–2.0)
	60–69	46.9	83.8	36.9	1.8	(1.4–2.4)	78.1	111.6	33.5	1.4	(1.1–1.8)
	70–79	67.2	73.6	6.4	1.1	(0.7–1.7)	94.6	93.0	−1.6	1.0	(0.7–1.4)
Mitotic count (mitoses/mm^2^)											
≤3.6	40–49	17.8	45.4	27.6	2.5	(1.5–4.2)	31.6	66.5	34.9	2.1	(1.4–3.2)
	50–59	20.0	101.1	81.1	5.0	(3.5–7.3)	43.2	140.6	97.4	3.3	(3.4–4.4)
	60–69	60.9	163.4	102.5	2.7	(2.1–3.4)	90.5	211.9	121.4	2.3	(1.9–2.9)
	70–79	110.0	138.7	28.7	1.2	(0.9–1.6)	145.3	166.6	21.3	1.1	(0.9–1.5)
> 3.6	40–49	14.9	40.3	25.4	2.7	(1.6–4.6)	46.4	60.9	14.5	1.3	(0.9–1.9)
	50–59	34.6	51.4	16.8	1.5	(1.1–2.0)	66.5	73.8	7.3	1.1	(0.8–1.5)
	60–69	48.8	65.9	17.1	1.3	(1.0–1.8)	80.0	86.4	6.4	1.1	(0.8–1.4)
	70–79	73.8	59.4	−14.4	0.8	(0.5–1.3)	101.1	73.7	−27.4	0.7	(0.5–1.1)
< 7.7	40–49	26.8	66.4	39.6	2.5	(1.7–3.7)	53.6	98.6	45	1.8	(1.3–2.5)
	50–59	38.3	132.0	93.7	3.5	(2.6–4.5)	78.8	185.4	106.6	2.4	(1.9–2.9)
	60–69	90.4	198.4	108	2.2	(1.8–2.6)	136.6	258.3	121.7	1.9	(1.6–2.2)
	70–79	160.0	181.2	21.2	1.1	(0.9–1.5)	203.5	216.6	13.1	1.1	(0.8–1.3)
≥7.7	40–49	5.9	19.3	13.4	3.2	(1.4–7.5)	24.3	28.8	4.05	1.2	(0.7–2.1)
	50–59	16.4	20.5	4.1	1.2	(0.8–2.0)	30.9	28.9	−2	0.9	(0.6–1.5)
	60–69	19.3	30.8	11.5	1.6	(1.0–2.5)	33.8	40.0	6.2	1.2	(0.8–1.9)
	70–79	29.9	17.0	−12.9	0.6	(0.2–1.3)	43.0	23.7	−19.3	0.6	(0.3–1.2)

^a^ Based on 50 imputed datasets using age (5-year categories) and calendar year of diagnosis (continuous), stage (I, II, III, IV, unknown) and extent of disease (disease localized to the breast, local invasion, regional lymph nodes, distant lymph nodes or organ metastases, unknown) as reported by the Cancer Registry of Norway, year of birth (5-year categories), observation time after diagnosis (log-transformed) and survival status (alive, death from breast cancer, death from other causes)

^b^ Breast cancer incidence from the Cancer Registry of Norway, including cases with unknown Ki-67-status and mitotic count

Abbreviations: *IRD* incidence rate difference, *IRR* incidence rate ratio, *CI* confidence interval

**Fig. 1:**
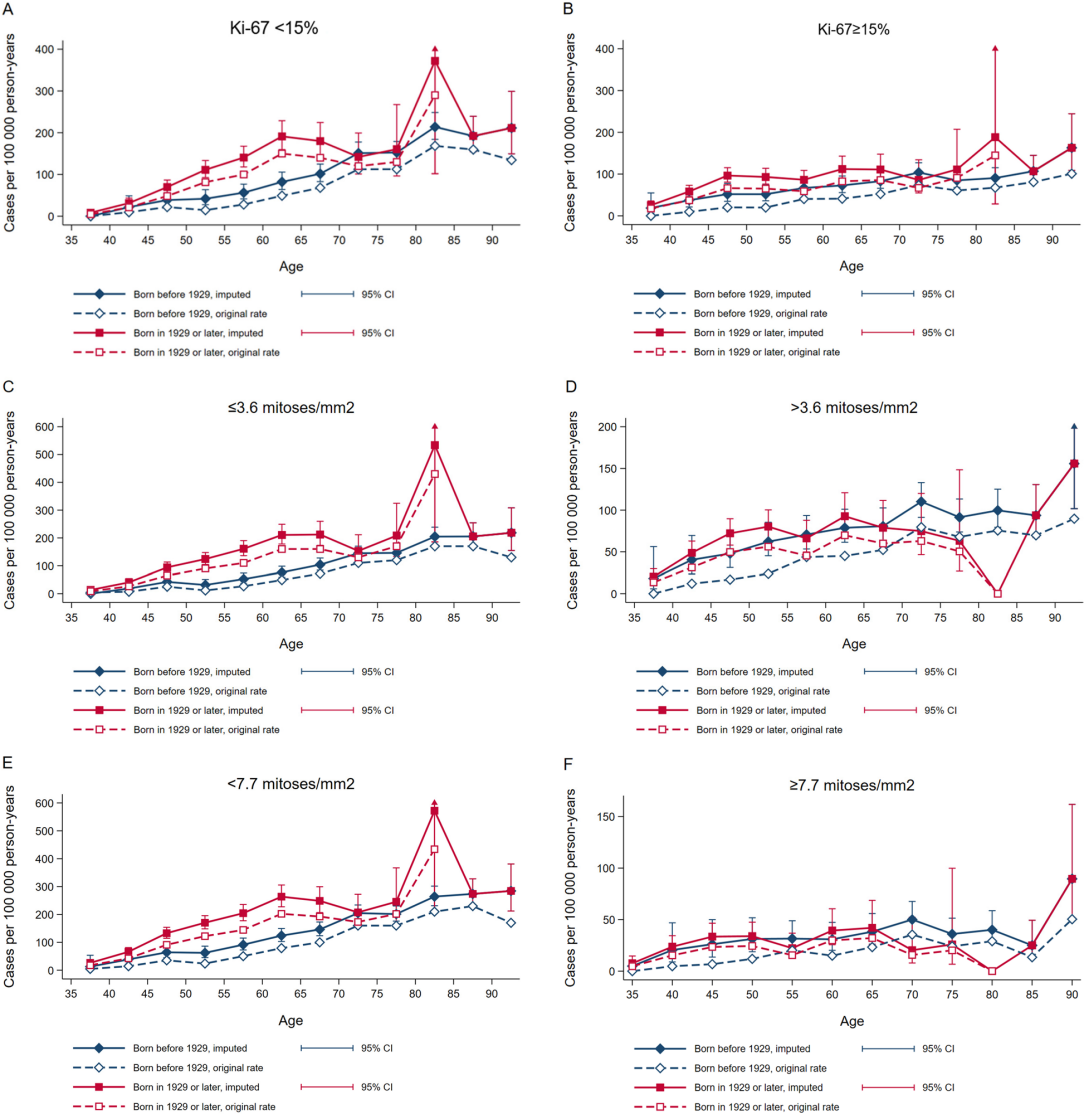
Incidence rates according to age, years of birth and proliferative marker status. Blue lines: women born before 1929. Red lines: Women born in 1929 or later. Dotted lines (red and blue) represent incidence rates of observed cases. Solid lines (red and blue) represent average incidence rates based on 50 imputed datasets with corresponding 95% CI. Fig. A-F shows breast cancer incidence according to A) Ki-67 < 15%, B) Ki-67 ≥ 15%, C) ≤3.6 mitoses/mm^2^, D) > 3.6 mitoses/mm^2^, E) < 7.7 mitoses/ mm^2^ and F) ≥7.7 mitoses/ mm^2^

Rates based on observed and imputed proliferation status followed the same patterns, with imputed rates being higher than observed rates. Imputed rates showed that the incidence of tumours with Ki-67 < 15% and Ki-67 ≥ 15% in the ages 40–69 years was higher among women born in 1929 or later, compared to women born before 1929 (Table 3, Fig. 1A and B). In the age group 70–79 years, there was no clear difference in breast cancer incidence according to Ki-67 at </≥15% cut-off. Using Ki-67 </≥30% as cut-off, we found an increase in imputed incidence rates of tumours with Ki-67 < 30% and ≥ 30% among women aged 40–69 years, but there was no clear difference in the age group 70–79 years (Supplementary Table 2 and Supplementary Fig. 2A and B).

For mitotic count, imputed values showed that the incidence rates of tumours with ≤3.6 mitoses/mm^2^ and < 7.7 mitoses/mm^2^ were higher in the age groups 40–69 years among women born in 1929 or later compared to women born before 1929 (Table 3, Fig. 1C, Fig. 1E). There was no clear difference in incidence rates of tumours with > 3.6 mitoses/mm^2^ or ≥ 7.7 mitoses/mm^2^ when comparing women born before 1929 to women born in 1929 or later (Table 3, Fig. 1D, Fig. 1F). Differences in incidence rates when comparing women born in 1929 or later to women born before 1929 according to both Ki-67 and mitotic count are given in Table 3.

### Mean values of proliferation markers according to age and birth year

We compared age-specific mean values for Ki-67 and mitotic count in women born before 1929 and women born in 1929 or later (Table 4). Differences in mean values according to birth cohort varied depending on age. Our analyses indicated that in the age group < 49 years mean values of both Ki-67 and mitotic counts were higher among women born in 1929 or later compared to women born before 1929. In the age groups 50–64 and 70–74 years according to Ki-67 and in all aged > 50 years according to mitotic count mean values were lower among women born in 1929 or later compared to those born before 1929.

**Table 4:** Mean Ki-67 (%) and mean mitoses/mm^2^ according to birth year and age at diagnosis

	Birth year in categories			
	Born before 1929		Born in 1929 or later	
Age	Mean Ki-67 (%) (SD)	Cases (n)	Mean Ki-67 (%) (SD)	Cases (n)
< 45	14.9 (13.6)	8	27.5 (20.1)	133
45–49	16.8 (12.2)	25	22.6 (18.9)	166
50–54	24.7 (19.1)	26	19.2 (19.1)	180
55–59	23.2 (17.2)	60	15.9 (15.7)	156
60–64	19.0 (16.7)	91	16.1 (16.1)	163
65–69	16.2 (14.4)	129	16.5 (18.0)	105
70–74	16.4 (15.9)	215	14.9 (15.0)	47
75–79	13.9 (12.2)	191	16.2 (20.0)	22
80–84	13.7 (12.9)	206	15.7 (6.2)	3
Age	Mean mitoses/mm^2^ (SD)	Cases (n)	Mean mitoses/mm^2^ (SD)	Cases (n)
< 45	6.3 (6.4)	8	6.7 (8.5)	132
45–49	4.1 (4.6)	25	5.5 (7.1)	166
50–54	7.5 (8.5)	27	4.7 (6.7)	180
55–59	6.2 (6.0)	62	3.1 (4.1)	156
60–64	5.3 (6.7)	95	3.7 (5.3)	163
65–69	4.6 (5.9)	133	4.1 (6.4)	105
70–74	5.0 (7.7)	222	3.4 (4.0)	47
75–79	3.7 (5.0)	202	2.7 (3.8)	22
80–84	3.0 (4.4)	213	1.5 (3.2)	3

Abbreviations: *SD* standard deviation

### Time trends in proliferative status within triple negative and HER2^+^ tumours

We compared incidence rates of HER2^+^ and TN tumours according to birth year, Ki-67-status, and mitotic count (Table 5). For HER2^+^ tumours, we found that incidence rates of tumours with Ki-67 ≥ 15% increased from 11.4/100000 person-years among women born before 1929 to 17.2/100000 person-years among women born in 1929 or later (HR 1.6, 95% CI 1.1–2.4). The incidence rate of HER2^+^ tumours with Ki-67 ≥ 30% was also higher among women born in 1929 or later, compared to women born before 1929 (HR 2.1 (95% CI 1.3–3.6)). For HER2^+^ tumours with ≤3.6 mitoses/mm^2^ incidence rates increased among women born in 1929 or later, compared to women born before 1929 (HR 1.9 (95% CI 1.1–3.2)). We found no clear changes in incidence rates among TN tumours.

**Table 5:** Incidence rates and hazard rates for subdivisions of molecular subtypes according to proliferation status and year of birth ^a^

Molecular subtype	Proliferation marker	Born before 1929	Born in 1929 or later	HR	95% CI
Ki-67 (%)					
HER2^+^	< 15	7.0	7.5	1.1	(0.7–1.9)
	≥15	11.4	17.2	1.6	(1.1–2.4)
Triple negative	< 15	3.7	2.7	1.1	(0.5–2.6)
	≥15	8.3	9.7	1.4	(0.9–2.3)
HER2^+^	< 30	13.2	14.8	1.2	(0.8–1.7)
	≥30	5.2	9.9	2.1	(1.3–3.6)
Triple negative	< 30	6.1	4.3	1.1	(0.6–2.2)
	≥30	5.8	8.0	1.5	(0.9–2.5)
Mitoses/mm^2^					
HER2^+^	≤3.6	6.0	9.7	1.9	(1.1–3.2)
	> 3.6	13.3	15.1	1.2	(0.8–1.7)
Triple negative	≤3.6	3.6	2.4	1.0	(0.4–2.4)
	> 3.6	9.3	10.0	1.3	(0.8–2.1)
HER2^+^	< 7.7	13.2	18.8	1.5	(1.0–2.1)
	≥7.7	6.2	5.9	1.1	(0.6–1.9)
Triple negative	< 7.7	7.5	5.1	1.1	(0.6–1.9)
	≥7.7	5.5	7.3	1.5	(0.8–2.5)

^a^ Analyses were limited to participants between 50 and 80 years

^b^ HER2^+^ comprises luminal B (HER2^+^) and HER2-type tumours

^c^ Trippel Negative comprises basal phenotype and 5 negative phenotype tumours

Abbreviations: *HR* hazard ratio, *CI* confidence interval

## Discussion

In this large population-based study of Norwegian women born between 1886 and 1977, age-specific breast cancer incidence rates were higher among women born in 1929 or later, compared to women born before 1929. We found an increase in incidence for both low- and high proliferative breast cancers, using Ki-67 as a marker of proliferation. According to mitotic count, we found an increase in breast cancers with low-proliferative status. However, there was no increase in high-proliferative tumours according to mitotic count. Even though we found an increase in both high- and low-proliferative tumours, the increase was most prominent for low-proliferative tumours. We also did separate incidence analyses for HER2^+^ and TN tumours. We found an increase in incidence of HER2^+^ tumours with Ki-67 ≥ 15% and Ki-67 ≥ 30%, while according to mitotic count, there was an increase in tumours with low mitotic count (≤3.6 mitoses/mm^2^). There was no change in breast cancer incidence according to proliferation status among TN tumours.

Since implementation of the mammography screening program in Norway attendance has been high [45]. Mammography screening favours detection of HER2 negative, luminal tumours [46, 47], particularly luminal A [46]. Population based mammography screening was introduced in the county of Trøndelag in 2001 for all women between 50 and 69 years of age [45]. In the present study, we compared women born in 1929 or later to women born before 1929. Since they were older than 69 years of age at the time of implementation, women born before 1929 were not included in the mammography screening program. The same cut-off for birth year was also used in a previous study by our group [7].

In the era of multigene assays, Ki-67 still plays a role as a predictive marker in breast cancer [36, 48, 49]. However, its use is debated because of inter- and intra-observer variations, laboratory variations, and the lack of consensus regarding cut-off-values, scoring and reporting [34, 50, 51]. Prior to the introduction of gene expression tests, Ki-67 cut-offs of 15 and 30% were used in the clinic to select patients for chemotherapy [52, 53]. In molecular subtyping, a TMA study by Cheang et al. found that 13.25% was the optimal Ki-67 cut-off to separate luminal A from luminal B (HER2^−^) tumours [12]. Subsequent 2011 St. Gallen guidelines defined 14% as the cut-off for Ki-67 to separate luminal A from luminal B tumours [54], even though clinical Ki-67 assessment is usually performed on whole sections. Knutsvik et al. have shown that Ki-67 values differ depending on specimen type, and that specimen-specific cut-off levels may be appropriate [55]. We used 15% as Ki-67 cut-off in molecular subtyping in the three cohorts included in this study. Since there is no consensus on Ki-67 cut-off levels, we used two different cut-offs in our incidence analyses, and selected two cut-offs that have been used both in the clinical setting, and for molecular subtyping: </≥15% and </≥30%.

A strength of this study is the large cohort of Norwegian women included, with long term follow-up for breast cancer occurrence. This gives us a unique opportunity to examine time trends in breast cancer incidence. Surveillance of disease patterns in a population over time is a corner stone in public health and often the first step in identification of previously unknown or emerging risk factors. Ki-67 immunostaining was performed in the same laboratory using the same IHC protocol and antibody for all cases. Mitotic count was evaluated independently by two pathologists, and Ki-67 was evaluated by two independent observers of whom at least one was a pathologist.

There are also some limitations to our study. The three cohorts included comprise women from the northern and southern parts of Trøndelag county. There is overlap in birth period between the cohorts, and some women were therefore included in more than one cohort. Breast cancer cases were identified through assigned case specific identity numbers, and we were able to identify overlapping incident cases. Due to anonymization of study participants, we could not identify overlap among the healthy background populations. Therefore, excluding overlapping incident breast cancer cases would have led to an underestimation of breast cancer incidence rates. Overlap in the healthy background population could have been avoided by removing participants with overlapping birth year, however this would lead to exclusion of more than half of the study population. We assumed that the overlap among women who developed breast cancer was proportionally similar to the overlap among the other study participants, and therefore did not exclude duplicate incident cancers in the incidence analysis. It could nevertheless lead to some overestimation of precision.

Information about Ki-67 and mitotic count was unavailable for some tumours, mainly because these patients were diagnosed at other hospitals. To compensate for the missing data, we used multiple imputations to prevent underestimation of incidence rates. Our imputation model included all available data, such as year of birth, age and calendar year at diagnosis, stage and extent of disease, follow up time after diagnosis and survival status. Even though such clinical information from national registries was included in the imputation model to prevent biased results, it is difficult to assess how well the imputed rates reflects the true values. However, observed and imputed incidence rates followed the same age-specific patterns, and although weaker, we found that the differences in incidence rates persisted after imputation.

The study was performed on archival tissue from six decades; hence preanalytical conditions may have varied. Studies of Ki-67 antigenicity after storage of tissue blocks have shown discrepant results [56, 57]. We found high Ki-67 levels in tumour tissue across all storage periods, and higher incidence of low-proliferative incident breast cancers among women born in 1929 or later, compared to women born before 1929. Nevertheless, we cannot exclude a decrease in antigenicity as a result of prolonged storage in the oldest tumours. Reduced Ki-67 antigenicity could therefore have led to an underestimation of the increase in incidence of tumours with low proliferative activity in women born in 1929 or later, and a false relative increase in tumours with high proliferative activity in women born in 1929 or later. Tissue storage does not influence the number of mitoses, and according to mitotic count we only found an increase in tumours with low proliferative activity. Thus, the increase in high proliferative tumours according to Ki-67 may in part be explained by loss of Ki-67 antigenicity over time.

Several studies have found increasing incidence rates of ER^+^ tumours and decreasing rates of ER^−^ tumours [14–18]. Hormone receptor positive tumours are in general less proliferative than hormone receptor negative tumours [11, 31], and they can be further subdivided into luminal A and luminal B based on their proliferative status [10, 35]. We have previously demonstrated increasing incidence rates for the luminal subtypes, especially luminal A [7]. Hence, our findings of increased incidence of tumours with low proliferative activity are in accordance with previously described incidence trends. To examine a possible change in proliferation within the molecular subtypes we made separate incidence analyses among HER2^+^ and TN tumours. For HER2^+^ tumours we found an increase of tumours with high proliferative status according to Ki-67, while according to mitotic count there was an increase of tumours with low proliferative status. We found no difference in incidence of TN tumours according to proliferation status. The observed increase in tumours with high-proliferative activity according to Ki-67 while not according to mitotic count could partly be explained by reduced Ki-67 antigenicity in the oldest set of tumours due to storage. Furthermore, Ki-67 is expressed during all phases of the cell cycle except G0, while mitotic figures only occur during the M-phase [58, 59]. Discrepant levels of Ki-67 and mitotic count have been described [60], and could explain the difference in incidence rates according to proliferative markers. Additionally, we assessed Ki-67 on TMAs while mitotic count was assessed on whole sections. Even though good correlation between TMAs and whole sections has been demonstrated [61], intratumoural heterogeneity may be a challenge.

The increase in low-proliferative breast cancers over time may partly be explained by the introduction of mammography screening programs and MHT [47, 62, 63]. However, increasing incidence over time for breast cancers with low-proliferative activity among women aged 40–49 years cannot be explained by mammography screening or MHT. Reproductive factors such as low parity, early menarche and late menopause are mainly associated with increased risk of luminal A tumours [4, 62]. Risk factors associated with the other molecular subtypes are not yet fully understood, and studies have shown inconsistent results [6, 62]. The observed changes in breast cancer incidence according to proliferation status in our study are most likely multifactorial and may be affected by both reproductive factors and lifestyle factors, such as obesity and alcohol consumption [4, 6, 64].

In conclusion, there has been an increase in incidence rates of tumours with low-proliferative activity, measured by Ki-67 and by mitotic count. We also found an increase in tumours with high-proliferative activity according to Ki-67, but not according to mitotic count.

## Supplementary Information

**Additional file 1.** Supplementary Fig. 1. Graphical display of birth year, age distribution and follow up period for the three cohorts. Cohort 1 is marked in yellow, cohort 2 in orange and cohort 3 in blue. Age according to birth year and year of follow up is listed in the background. Supplementary Fig. 2. Incidence rates according to age, years of birth and proliferative marker status. Blue lines: women born before 1929. Red lines: Women born in 1929 or later. Dotted lines (red and blue) represent incidence rates of observed cases. Solid lines (red and blue) represent average incidence rates based on 50 imputed datasets with corresponding 95% CI

**Additional file 2.** Supplementary Table 1. Characteristics of breast cancer patients according to Ki-67-status (missing vs. non-missing). Supplementary Table 2. Incidence rates, incidence rate differences (IRD) and incidence rate ratios (IRR) of proliferation marker Ki-67

## Data Availability

The data that support the findings of this study are not openly available due to sensitive personal data. However, anonymized data may be available from Professor Anna Mary Bofin (anna.bofin@ntnu.no) upon request and with permission of the regional ethics committee

## Abbreviations

BP: Basal phenotype
CI: Confidence interval
CISH: Chromogenic in situ hybridization
ER: Oestrogen receptor
FDA: Food and Drugs Administration
FFPE: Formalin-fixed paraffin-embedded
FISH: Fluorescence in situ hybridization
HER2: Human epidermal growth factor receptor 2
HR: Hazard ratio
IHC: Immunohistochemistry
IRD: Incidence rate difference
IRR: Incidence rate ratio
ISH: In situ hybridization
MHT: Menopausal hormone therapy
SD: Standard deviation
TMA: Tissue micro array
TN: Triple negative
