# Supplementary material for: Long term trends of breast cancer incidence according to proliferation status
Source: BMC Cancer. 2022 Dec 21;22:1340. doi: 10.1186/s12885-022-10438-1 (PMC9773605; doi:10.1186/s12885-022-10438-1)
Supplement: Supplementary file 1 — Additional file 1. Supplementary Fig. 1. Graphical display of birth year, age distribution and follow up period for the three cohorts. Cohort 1 is marked in yellow, cohort 2 in orange and cohort 3 in blue. Age according to birth year and year of follow up is listed in the background. Supplementary Fig. 2. Incidence rates according to age, years of birth and proliferative marker status. Blue lines: women born before 1929. Red lines: Women born in 1929 or later. Dotted lines (red and blue) represent incidence rates of observed cases. Solid lines (red and blue) represent average incidence rates based on 50 imputed datasets with corresponding 95% CI [file 12885_2022_10438_MOESM1_ESM.zip › Supplementary figure 1_med figurtekst.docx]

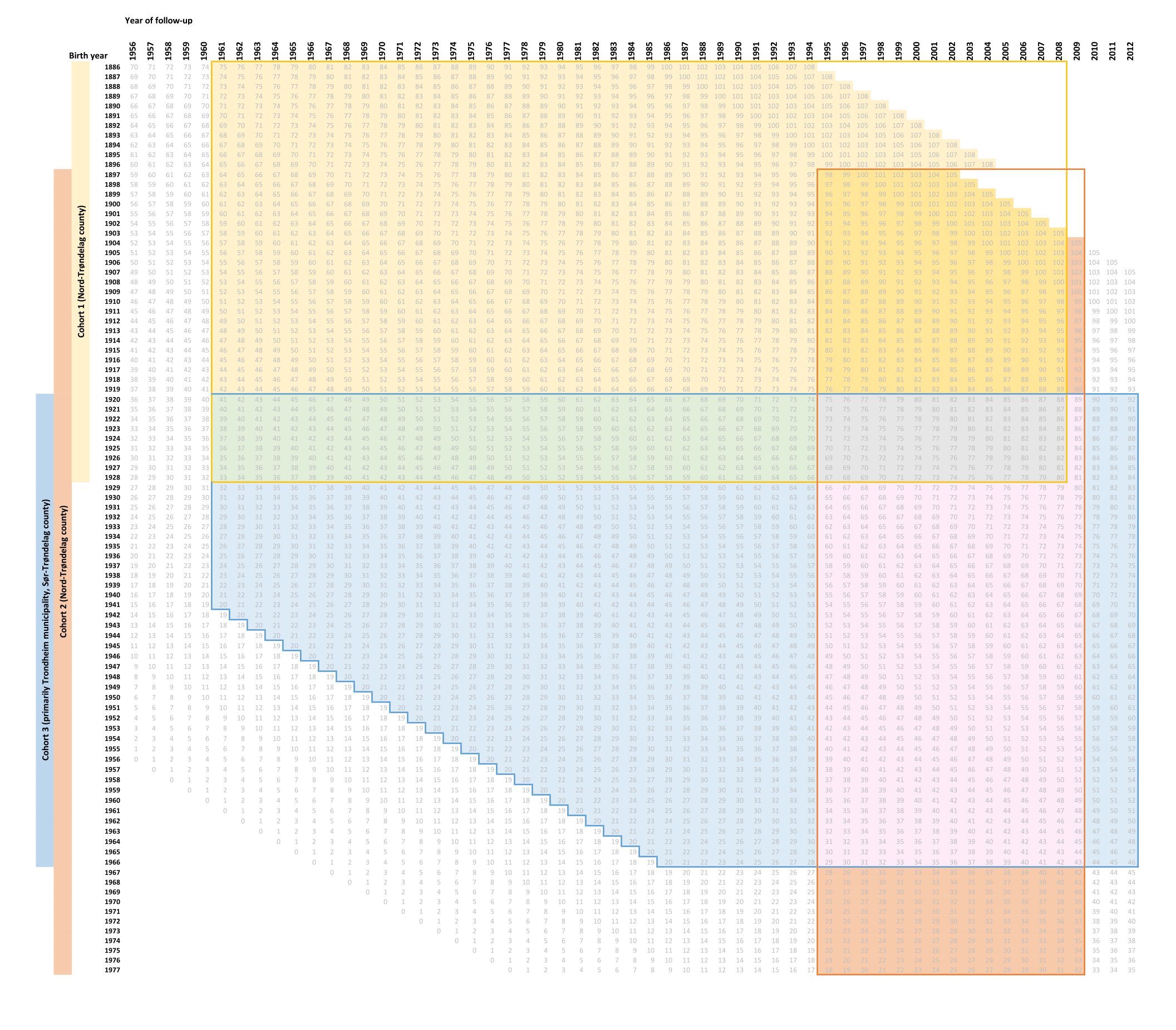
**Supplementary figure 1:** Graphical display of birth year, age distribution and follow up period for the three cohorts. Cohort 1 is marked in yellow, cohort 2 in orange and cohort 3 in blue. Age according to birth year and year of follow up is listed in the background.
