# Supplementary material for: Long term trends of breast cancer incidence according to proliferation status
Source: BMC Cancer. 2022 Dec 21;22:1340. doi: 10.1186/s12885-022-10438-1 (PMC9773605; doi:10.1186/s12885-022-10438-1)
Supplement: Supplementary file 2 — Additional file 2. Supplementary Table 1. Characteristics of breast cancer patients according to Ki-67-status (missing vs. non-missing). Supplementary Table 2. Incidence rates, incidence rate differences (IRD) and incidence rate ratios (IRR) of proliferation marker Ki-67 [file 12885_2022_10438_MOESM2_ESM.zip › Supplementary table 1.docx]

| **Supplementary table 1:** Characteristics of breast cancer patients according to Ki-67-status (missing vs. non-missing) | | | | |
| --- | --- | --- | --- | --- |
|  | **Born before 1929**  **(n=1641)** | | **Born in 1929 or later (n=1354)** | |
|  | **Non-missing** | **Missing** | **Non-missing** | **Missing** |
| Number of women (%) | 1100 (67) | 541 (33) | 975 (72) | 379 (28) |
| Mean age at baseline (SD) ^a^ | 50.5 (13.8) | 52.1 (12.5) | 35.1 (15.8) | 25 (10.9) |
| Mean age at diagnosis (SD) | 73.5 (10.6) | 68.0 (13.1) | 55.1 (9.8) | 53 (9.6) |
| Mean year of diagnosis (SD) | 1989 (11.7) | 1979 (13.0) | 2002 (6.5) | 2002 (7.3) |
| Mean follow up for BC occurrence (SD) | 23.4 (12.8) | 16.5 (12.1) | 20.5 (14.4) | 28.5 (12.1) |
| Death from breast cancer (%) | 427 (39) | 271 (50) | 145 (15) | 65 (17) |
| Death from other causes (%) | 570 (52) | 246 (45) | 82 (8) | 23 (6) |
| **Stage (%)** ^b^ |  |  |  |  |
| I | 521 (47) | 255 (47) | 410 (42) | 151 (40) |
| II | 426 (39) | 157 (29) | 336 (35) | 72 (19) |
| III | 71 (6) | 38 (7) | 34 (3) | 6 (2) |
| IV | 54 (5) | 73 (14) | 23 (2) | 22 (6) |
| Unknown | 28 (3) | 18 (3) | 172 (17) | 128 (34) |
| **Extent of disease (%)** ^b^ |  |  |  |  |
| Disease localized to the breast | 377 (34) | 217 (40) | 499 (51) | 202 (53) |
| Local invasion | 37 (3) | 14 (3) | 6 (1) | 0 |
| Regional lymph nodes | 292 (27) | 148 (27) | 344 (35) | 101 (27) |
| Distant lymph node or organ metastases | 43 (4) | 68 (13) | 20 (2) | 22 (6) |
| Metastases detected, unknown location | 0 | 2 (0) | 0 | 0 |
| Unknown | 351 (32) | 92 (17) | 106 (11) | 54 (14) |
| ^a)^  At time of entry  ^b)^ As recorded by the Cancer registry of Norway. Information is based on histopathological and/or clinical examination.  Abbreviations: *SD* standard deviation | | | | |
