# Supplementary material for: Long term trends of breast cancer incidence according to proliferation status
Source: BMC Cancer. 2022 Dec 21;22:1340. doi: 10.1186/s12885-022-10438-1 (PMC9773605; doi:10.1186/s12885-022-10438-1)
Supplement: Supplementary file 2 — Additional file 2. Supplementary Table 1. Characteristics of breast cancer patients according to Ki-67-status (missing vs. non-missing). Supplementary Table 2. Incidence rates, incidence rate differences (IRD) and incidence rate ratios (IRR) of proliferation marker Ki-67 [file 12885_2022_10438_MOESM2_ESM.zip › Supplementary table 2.docx]

| **Supplementary table 2:** Incidence rates, incidence rate differences (IRD) and incidence rate ratios (IRR) of proliferation marker Ki-67 | | | | | | | | | | | |
| --- | --- | --- | --- | --- | --- | --- | --- | --- | --- | --- | --- |
|  | | **Observed** | | | | | **Imputed^a^** | | | | |
|  | | **Incidence rate**  **(cases/100 000 person-years)** | |  | | | **Incidence rate**  **(cases/100 000 person-years)** | |  | | |
| **Ki-67(%)** | **Age** | **Women born before 1929** | **Women born in**  **1929 or later** | **IRD** | **IRR** | **(95% CI)** | **Women born before 1929** | **Women born in**  **1929 or later** | **IRD** | **IRR** | **(95% CI)** |
| **<30** | 40-49 | 27.8 | 60.7 | 32.90 | 2.2 | (1.5-3.3) | 58.4 | 89.7 | 31.30 | 1.5 | (1.1-2.1) |
|  | 50-59 | 38.9 | 124.3 | 85.40 | 3.2 | (2.4-4.1) | 83.5 | 173.4 | 89.90 | 2.1 | (1.7-2.6) |
|  | 60-69 | 88.4 | 193.3 | 104.90 | 2.2 | (1.8-2.6) | 140.8 | 248.9 | 108.10 | 1.8 | (1.5-2.1) |
|  | 70-79 | 159.1 | 167.0 | 7.90 | 1.0 | (0.8-1.4) | 215.1 | 202.8 | -12.30 | 0.9 | (0.7-1.2) |
| **≥30** | 40-49 | 5.0 | 25.4 | 20.40 | 5.1 | (2.1-12.7) | 19.1 | 37.6 | 18.50 | 2.0 | (1.0-3.9) |
|  | 50-59 | 14.0 | 28.2 | 14.20 | 2.0 | (1.3-3.3) | 26.3 | 40.8 | 14.50 | 1.6 | (1.0-2.4) |
|  | 60-69 | 17.4 | 36.8 | 19.40 | 2.1 | (1.4-3.3) | 29.6 | 49.5 | 19.90 | 1.7 | (1.1-2.5) |
|  | 70-79 | 20.7 | 28.3 | 7.60 | 1.4 | (0.7-2.7) | 31.3 | 37.5 | 6.20 | 1.2 | (0.6-2.3) |
| ^a^ Based on 50 imputed datasets using age (5-year categories) and calendar year of diagnosis (continous), stage (I, II, III, IV, unknown) and extent of disease (disease localized to the breast, local invasion, regional lymph nodes, distant lymph nodes or organ metastases, unknown) as reported by the Cancer Registry of Norway, year of birth (5-year categories), observation time after diagnosis (log-transformed) and survival status (alive, death from breast cancer, death from other causes).  ^b^ Breast cancer incidence from the Cancer Registry of Norway, including cases with unknown Ki-67-status and mitotic count  Abbreviations: *IRR* incidence rate ratios, *CI* confidence interval, | | | | | | | | | | | |
